# Clinical Profile, Management Patterns, and In-Hospital Outcomes Among Patients Hospitalized With Atrial Fibrillation at a Tertiary Care Hospital: A Single-Center Retrospective Study

**DOI:** 10.7759/cureus.112990

**Published:** 2026-07-20

**Authors:** Ali Husnain, Muhammad Tausif Khan, Sofia Salman Sabir, Shahryar Shaukat, Amna Rehman, Faisal Shahzad, Sadia Khan

**Affiliations:** 1 Cardiology, Shalamar Hospital, Lahore, PAK; 2 Internal Medicine, Federal Government PolyClinic Hospital, Islamabad, PAK; 3 Internal Medicine, Northwest General Hospital and Research Center, Peshawar, PAK; 4 Psychiatry, ABWA Medical College, Faisalabad, PAK; 5 Internal Medicine, Islamabad Medical and Dental College, Islamabad, PAK; 6 Internal Medicine, Shaikh Zayed Hospital, Rahim Yar Khan, PAK; 7 Internal Medicine, Sindh Government, Lyari General and Teaching Hospital, Karachi, PAK

**Keywords:** anticoagulation, atrial fibrillation, clinical profile, comorbidities, hospitalized patients

## Abstract

Background: Atrial fibrillation (AF) is the most common sustained cardiac arrhythmia worldwide and is associated with substantial morbidity, mortality, recurrent hospitalization, and healthcare expenditure.

Objective: This study aimed to evaluate the clinical characteristics, management patterns, and in-hospital outcomes of patients hospitalized with AF and to identify factors associated with in-hospital mortality.

Methods: This retrospective observational study included consecutive adult patients (≥18 years) with electrocardiographically confirmed AF admitted to Shalamar Hospital, Lahore, Pakistan, between January 1, 2023, and January 1, 2026. Demographic characteristics, comorbidities, echocardiographic findings, treatment modalities, and in-hospital outcomes were extracted from medical records. The primary outcome was in-hospital mortality. Secondary outcomes included stroke, systemic embolism, major bleeding, heart failure exacerbation, intensive care unit admission, and length of hospital stay. Univariable and exploratory multivariable logistic regression analyses were performed, and adjusted odds ratios (ORs) with 95% confidence intervals (CIs) were reported.

Results: A total of 282 patients were included (mean age, 68.4 ± 11.9 years; 58.5% male patients). Hypertension (70.2%), diabetes mellitus (44.7%), ischemic heart disease (40.4%), and heart failure (34.8%) were the most common comorbidities. Persistent AF was the most frequent subtype (36.5%), while paroxysmal and permanent AF comprised 29.8% and 33.7% of cases, respectively. Rate-control therapy was administered to 88.3% of patients, and anticoagulation to 80.5% (direct oral anticoagulants 52.5%; warfarin 28.0%). In-hospital mortality was 5.7%. Exploratory multivariable analysis demonstrated that age ≥75 years (adjusted OR, 2.48; 95% CI, 1.08-5.71; p = 0.032), heart failure (adjusted OR, 2.91; 95% CI, 1.24-6.82; p = 0.014), chronic kidney disease (adjusted OR, 3.67; 95% CI, 1.49-9.01; p = 0.005), and LVEF <40% (adjusted OR, 2.74; 95% CI, 1.17-6.41; p = 0.020) were associated with in-hospital mortality.

Conclusion: Hospitalized patients with AF were predominantly older adults with multiple cardiovascular comorbidities and experienced considerable in-hospital morbidity despite contemporary management. Advanced age, heart failure, chronic kidney disease, and reduced left ventricular ejection fraction were associated with in-hospital mortality. These findings may help identify patients requiring closer inpatient monitoring and optimized evidence-based management; however, larger prospective multicenter studies are needed to validate these associations.

## Introduction

Atrial fibrillation (AF) is the most common sustained cardiac arrhythmia encountered in clinical practice and represents a major global public health challenge. AF is associated with increased morbidity, mortality, reduced quality of life, and substantial healthcare expenditure, largely because of its thromboembolic complications, heart failure, and coexistence with multiple cardiovascular comorbidities rather than the arrhythmia itself [1]. Population aging, improved survival from cardiovascular disease, and the increasing prevalence of risk factors such as hypertension, obesity, diabetes mellitus, and heart failure have contributed to the growing global burden of AF [2]. Recent estimates indicate that more than 60 million people worldwide are living with AF, and this number is projected to more than double by 2060 owing to aging populations and improved screening and diagnostic strategies [3]. AF develops through complex structural, electrical, and autonomic remodeling of the atria, including atrial fibrosis, inflammation, oxidative stress, and atrial cardiomyopathy, which promote the initiation and maintenance of the arrhythmia. AF commonly coexists with hypertension, coronary artery disease, valvular heart disease, cardiomyopathies, heart failure, hyperthyroidism, chronic kidney disease, and obstructive sleep apnea [4]. Increasing age remains the strongest independent risk factor for AF, particularly after 65 years, while smoking, alcohol consumption, obesity, and physical inactivity further increase disease risk [5].

AF is classified as paroxysmal, persistent, long-standing persistent, or permanent. Clinical presentation ranges from asymptomatic disease to palpitations, dyspnea, chest pain, dizziness, syncope, fatigue, and hemodynamic instability [6]. Hospitalization commonly occurs because of rapid ventricular response, acute decompensated heart failure, thromboembolic complications, bleeding, or acute exacerbations of underlying cardiovascular disease and other medical conditions that either precipitate or coexist with AF [7]. Stroke remains one of the most serious complications of AF, primarily resulting from thrombus formation within the left atrial appendage. Although the magnitude of stroke risk varies with individual patient characteristics, AF substantially increases the risk of ischemic stroke, which is routinely assessed using the CHA₂DS₂-VASc score, whereas bleeding risk should be evaluated using validated tools such as HAS-BLED to guide anticoagulation decisions [8,9]. AF is also associated with heart failure progression, systemic embolism, cognitive impairment, recurrent hospitalization, and increased mortality. Furthermore, AF-related ischemic strokes are generally associated with greater neurological disability, higher mortality, and increased healthcare utilization than strokes unrelated to AF [10]. Emerging evidence also suggests that occult or previously undiagnosed AF contributes significantly to cryptogenic stroke, emphasizing the importance of early rhythm detection and risk stratification in susceptible patients [11].

Hospitalizations related to AF continue to increase worldwide and represent a major burden on healthcare systems, particularly among older adults with multiple comorbidities [12]. Optimal inpatient management requires prompt stabilization of acute triggers together with individualized long-term strategies for stroke prevention and symptom control [13]. Contemporary management includes anticoagulation, rate control, rhythm control (including electrical cardioversion and catheter ablation), and aggressive modification of cardiovascular risk factors [14]. Direct oral anticoagulants (DOACs) have largely replaced vitamin K antagonists for many patients with nonvalvular AF because they provide at least comparable efficacy for stroke prevention with a more favorable bleeding profile in appropriately selected patients. Nevertheless, treatment decisions remain complex in hospitalized patients because of advanced age, multiple comorbidities, varying thromboembolic and bleeding risks, and the need to balance acute management with long-term therapeutic goals. Advanced age, heart failure, chronic kidney disease, diabetes mellitus, hypertension, previous stroke, and reduced left ventricular systolic function have consistently been associated with adverse hospital outcomes [15]. Despite the increasing burden of AF, data describing the clinical characteristics, management patterns, and in-hospital outcomes of hospitalized patients from Pakistan remain limited. Understanding these patterns in a tertiary-care setting may help identify high-risk patients, evaluate current management practices, and guide strategies to improve in-hospital outcomes, including mortality, stroke, systemic embolism, major bleeding, intensive care unit (ICU) admission, and length of hospital stay [16].

Objective

This study aimed to evaluate the clinical characteristics, management patterns, and in-hospital outcomes of patients hospitalized with AF and to identify factors associated with in-hospital mortality.

## Materials and methods

Methodology

This retrospective observational study was conducted at Shalamar Hospital, Lahore, Pakistan, from January 1, 2023, to January 1, 2026. Owing to the retrospective review of anonymized medical records, the requirement for informed consent was waived by the Institutional Review Board.

Consecutive sampling, a nonprobability sampling technique, was employed to recruit all eligible patients during the study period. The study included 282 adult patients (≥18 years) admitted to the emergency department, cardiology service, medical wards, or ICU with AF confirmed by a standard 12-lead electrocardiogram (ECG). Both newly diagnosed and previously known AF cases were eligible.

Patients with incomplete clinical or outcome data, uncertain AF diagnosis, duplicate admissions, or transient perioperative AF occurring within the immediate postoperative period and resolving without requiring ongoing AF-specific management were excluded. Transient perioperative AF was identified retrospectively from operative records, ECG findings, discharge summaries, and treating physician documentation.

Data collection

Data were extracted retrospectively from electronic and paper medical records using a structured, pilot-tested data extraction proforma by two independent reviewers. Any discrepancies were resolved by consensus through review of the original medical records.

Demographic variables included age, sex, body mass index, smoking status, and place of residence. Clinical variables included presenting symptoms, symptom duration, AF subtype (paroxysmal, persistent, or permanent), and comorbid conditions including hypertension, diabetes mellitus, ischemic heart disease, heart failure, valvular heart disease, chronic kidney disease, chronic obstructive pulmonary disease, thyroid disorders, and previous stroke or transient ischemic attack (TIA). Initial vital signs recorded at hospital presentation (heart rate, systolic blood pressure, and other admission observations) were used for analysis. Electrocardiographic findings and echocardiographic parameters, including left ventricular ejection fraction (LVEF) and left atrial diameter, together with treatment details, were extracted from the medical records.

Treatment variables were categorized into acute inpatient management and discharge therapy. Acute inpatient management included rate-control therapy, rhythm-control therapy, pharmacological cardioversion, electrical cardioversion, antiarrhythmic therapy, anticoagulation, and admission to the ICU when indicated. Electrical cardioversion was performed for patients with hemodynamic instability or persistent symptomatic AF after clinical assessment according to institutional practice, whereas pharmacological cardioversion was used in selected hemodynamically stable patients. Discharge medications, including anticoagulation therapy, were recorded separately.

Hospital outcomes included length of hospital stay, worsening heart failure, ischemic stroke, systemic embolism, major bleeding, ICU admission, discharge status, and in-hospital mortality. Patients with incomplete demographic, treatment, or outcome data were excluded. No imputation of missing values was performed.

Outcome definitions

The primary outcome of the study was in-hospital mortality. Secondary outcomes included length of hospital stay, heart failure exacerbation, ischemic stroke, systemic embolism, major bleeding, ICU admission, and discharge status.

Major bleeding was defined according to the International Society on Thrombosis and Haemostasis criteria as fatal bleeding, symptomatic bleeding in a critical organ, bleeding causing a fall in hemoglobin of ≥2 g/dL, or bleeding requiring transfusion of at least two units of packed red blood cells. Systemic embolism was defined as clinically or radiologically confirmed embolism involving the noncerebral systemic arterial circulation. Heart failure exacerbation was defined as worsening signs or symptoms of heart failure requiring escalation of medical therapy during hospitalization. ICU admission was defined as transfer to the ICU at any time during the index hospitalization. Length of hospital stay was calculated as the number of days from hospital admission to discharge or death. AF was confirmed by a standard 12-lead ECG and documented by the treating physician or cardiologist in the medical record. AF subtype (paroxysmal, persistent, or permanent) was classified according to the treating cardiologist's documentation in accordance with contemporary guideline definitions.

Data analysis

Data were entered and analyzed using the Statistical Package for the Social Sciences, version 29.0 (IBM Corp., Armonk, NY). Continuous variables were expressed as mean ± standard deviation, while categorical variables were presented as frequencies and percentages.

Categorical variables were compared using the chi-square test or Fisher's exact test, as appropriate, whereas the independent samples t-test was used to compare the means of continuous variables across different in-hospital outcome groups. Age ≥75 years was selected for mortality analysis because this threshold has established clinical relevance and was specified a priori.

Variables considered clinically relevant or demonstrating a p value of <0.10 in univariable analysis were entered into an exploratory multivariable logistic regression model to evaluate their association with in-hospital mortality. Results are reported as adjusted odds ratios (ORs) with 95% confidence intervals (95% CIs). Model calibration was assessed using the Hosmer-Lemeshow goodness-of-fit test, and overall model performance was evaluated using the omnibus test of model coefficients. A two-sided p value of ≤0.05 was considered statistically significant.

## Results

Data were collected from 282 patients; the mean age was 68.4 ± 11.9 years, indicating that the majority of patients were aged ≥60 years. The largest age group was 60-69 years with 89 patients (31.6%), followed by 70-79 years with 82 patients (29.1%). Male patients were more common, representing 165 patients (58.5%), while female patients were 117 (41.5%) (Table 1).

**Table 1:** Baseline demographic characteristics of hospitalized patients with atrial fibrillation (n = 282) Continuous variables are expressed as mean ± SD, while categorical variables are presented as frequency and percentage BMI: body mass index; SD: standard deviation

Variable	Value
Age (years), mean ± SD	68.4 ± 11.9
Age group, n (%)	
<60 years	68 (24.1)
60-69 years	89 (31.6)
70-79 years	82 (29.1)
≥80 years	43 (15.2)
Gender, n (%)	
Male	165 (58.5)
Female	117 (41.5)
BMI (kg/m²), mean ± SD	28.1 ± 4.6
Smoking status, n (%)	
Current/former smoker	102 (36.2)
Nonsmoker	180 (63.8)
Duration of symptoms (days), mean ± SD	5.8 ± 3.4

Palpitations were the most common presenting symptom, reported in 198 patients (70.2%), followed by dyspnea in 174 (61.7%), chest pain in 88 (31.2%), and dizziness or syncope in 52 (18.4%). Hypertension was the leading comorbidity, present in 198 patients (70.2%), followed by diabetes mellitus in 126 (44.7%), ischemic heart disease in 114 (40.4%), and heart failure in 98 (34.8%). Persistent AF was the most frequent type, seen in 103 patients (36.5%), followed by permanent AF in 95 (33.7%) and paroxysmal AF in 84 (29.8%) (Table 2).

**Table 2:** Clinical characteristics of hospitalized patients with AF (n = 282) Continuous variables are expressed as mean ± standard deviation, whereas categorical variables are presented as frequency and percentage AF: atrial fibrillation; BP: blood pressure; COPD: chronic obstructive pulmonary disease; LVEF: left ventricular ejection fraction; TIA: transient ischemic attack

Variable	Category	Value
Presenting symptoms, n (%)		
Palpitations	Present	198 (70.2)
Dyspnea	Present	174 (61.7)
Chest pain	Present	88 (31.2)
Dizziness/syncope	Present	52 (18.4)
Comorbidities, n (%)		
Hypertension	Present	198 (70.2)
Diabetes mellitus	Present	126 (44.7)
Ischemic heart disease	Present	114 (40.4)
Heart failure	Present	98 (34.8)
Valvular heart disease	Present	72 (25.5)
Previous stroke/TIA	Present	55 (19.5)
Chronic kidney disease	Present	48 (17.0)
COPD	Present	41 (14.5)
AF classification, n (%)		
AF type	Paroxysmal AF	84 (29.8)
	Persistent AF	103 (36.5)
	Permanent AF	95 (33.7)
Clinical and echocardiographic parameters, mean ± SD		
Heart rate (beats/minute)		118.6 ± 21.4
Systolic BP (mmHg)		134.8 ± 22.5
LVEF (%)		46.7 ± 11.8
Left atrial diameter (mm)		46.3 ± 7.1
LVEF category, n (%)	<40%	83 (29.4)
	≥40%	199 (70.6)

Rate control therapy was the most commonly used management strategy and was given to 249 patients (88.3%), while rhythm control therapy was used in 101 patients (35.8%). Anticoagulation therapy was prescribed to 227 patients (80.5%), with DOACs used in 148 (52.5%) and warfarin in 79 (28.0%) (Table 3). Patients could receive more than one treatment modality during hospitalization.

**Table 3:** Treatment patterns during hospitalization Patients may have received more than one treatment modality; therefore, percentages are not mutually exclusive Values are presented as frequency (percentage)

Treatment		Value
Rate-control therapy, n (%)		249 (88.3)
Rhythm-control therapy, n (%)		101 (35.8)
Anticoagulation therapy, n (%)	Direct oral anticoagulants	148 (52.5)
	Warfarin	79 (28.0)
Electrical cardioversion, n (%)		38 (13.5)

The mean hospital stay was 6.7 ± 3.5 days. Heart failure exacerbation was the most common in-hospital complication, occurring in 43 patients (15.2%). Stroke occurred in 12 patients (4.3%), major bleeding in 9 (3.2%), and systemic embolism in 4 (1.4%). In-hospital mortality was reported in 16 patients (5.7%), while 266 patients (94.3%) survived (Table 4).

**Table 4:** In-hospital outcomes Values are presented as frequency (percentage) or mean ± standard deviation SD: standard deviation; ICU: intensive care unit

Outcome	Value
Length of hospital stay (days), mean ± SD	6.7 ± 3.5
Heart failure exacerbation, n (%)	43 (15.2)
Stroke, n (%)	12 (4.3)
Major bleeding, n (%)	9 (3.2)
Systemic embolism, n (%)	4 (1.4)
ICU admission, n (%)	42 (14.9)
In-hospital mortality, n (%)	16 (5.7)

In-hospital mortality was significantly associated with age ≥75 years, heart failure, chronic kidney disease, previous stroke/TIA, and reduced ejection fraction <40%. Among patients who died, 11 (68.8%) were aged ≥75 years compared with 114 survivors (42.9%), with p = 0.041. Heart failure was present in 10 deaths (62.5%) vs. 88 survivors (33.1%), with p = 0.018. Chronic kidney disease showed the strongest association, present in seven deaths (43.8%) vs. 41 survivors (15.4%), with p = 0.006 (Table 5). Univariate analysis demonstrated associations between in-hospital mortality and age ≥75 years (χ² = 4.18, p = 0.041), heart failure (χ² = 5.60, p = 0.018), chronic kidney disease (χ² = 7.63, p = 0.006), previous stroke/TIA (χ² = 3.87, p = 0.049), and LVEF <40% (χ² = 6.02, p = 0.014) (Table 5).

**Table 5:** Factors associated with in-hospital mortality Chi-square (χ²) test was used for categorical variables; Fisher's exact test was applied when expected cell counts were <5. A p value of ≤0.05 was considered statistically significant LVEF: left ventricular ejection fraction; TIA: transient ischemic attack

Variable	Mortality (n = 16), n (%)	Survivors (n = 266), n (%)	χ² (or Fisher's exact)	p value
Age ≥75 years	11 (68.8)	114 (42.9)	4.18	0.041
Hypertension	13 (81.3)	185 (69.5)	1.00	0.318
Diabetes mellitus	9 (56.3)	117 (44.0)	0.87	0.351
Heart failure	10 (62.5)	88 (33.1)	5.60	0.018
Chronic kidney disease	7 (43.8)	41 (15.4)	7.63	0.006
Previous stroke/TIA	6 (37.5)	49 (18.4)	3.87	0.049
LVEF <40%	9 (56.3)	74 (27.8)	6.02	0.014

Exploratory multivariable logistic regression analysis demonstrated that age ≥75 years (adjusted OR: 2.48, 95% CI: 1.08-5.71, p = 0.032), heart failure (adjusted OR: 2.91, 95% CI: 1.24-6.82, p = 0.014), chronic kidney disease (adjusted OR: 3.67, 95% CI: 1.49-9.01, p = 0.005), and reduced LVEF (<40%) (adjusted OR: 2.74, 95% CI: 1.17-6.41, p = 0.020) were associated with in-hospital mortality. Given the limited number of mortality events (n = 16), these findings should be interpreted cautiously. Although previous stroke/TIA showed a trend toward higher mortality risk, the association did not reach statistical significance (adjusted OR: 2.12, 95% CI: 0.92-4.91, p = 0.077) (Table 6).

**Table 6:** Exploratory multivariable logistic regression analysis of factors associated with in-hospital mortality Variables with clinical relevance and/or p < 0.10 in univariable analysis were entered into the exploratory multivariable logistic regression model. Adjusted ORs with 95% CIs are reported. Given the limited number of in-hospital deaths (n = 16), the findings should be interpreted as exploratory. Wald χ² statistics were used to assess the significance of individual factors. A p value of ≤0.05 was considered statistically significant. Model goodness-of-fit was assessed using the omnibus test of model coefficients and the Hosmer-Lemeshow test LVEF: left ventricular ejection fraction; TIA: transient ischemic attack; OR: odds ratio; CI: confidence interval

Variable	β coefficient	Adjusted OR	95% CI	Wald χ²	p value
Age ≥75 years	0.91	2.48	1.08-5.71	4.59	0.032
Heart failure	1.07	2.91	1.24-6.82	6.06	0.014
Chronic kidney disease	1.30	3.67	1.49-9.01	8.02	0.005
Previous stroke/TIA	0.75	2.12	0.92-4.91	3.12	0.077
LVEF (<40%)	1.01	2.74	1.17-6.41	5.40	0.020

The exploratory multivariable logistic regression model demonstrated acceptable overall fit. The omnibus test of model coefficients was statistically significant (χ² = 29.84, df = 5, p < 0.001), indicating that the model provided a better fit than the intercept-only model. The Hosmer-Lemeshow goodness-of-fit test was nonsignificant (χ² = 5.72, df = 8, p = 0.679), suggesting adequate calibration. The Cox and Snell R² and Nagelkerke R² values were 0.17 and 0.31, respectively, indicating that the model explained a moderate proportion of the variability in in-hospital mortality. However, because only 16 mortality events occurred, the multivariable findings should be interpreted as exploratory and require validation in larger studies (Table 6).

## Discussion

This study evaluated the clinical profile, management patterns, and in-hospital outcomes of 282 patients hospitalized with AF at a tertiary care center. The findings demonstrate that the majority of patients were older adults and had multiple cardiovascular comorbidities, with hypertension being the most prevalent, followed by diabetes mellitus, ischemic heart disease, and heart failure. Persistent AF was the most common subtype, while rate-control therapy and anticoagulation constituted the predominant management strategies. Despite contemporary treatment, clinically important in-hospital complications, including heart failure exacerbation, stroke, major bleeding, and mortality, remained common.

The demographic characteristics observed in this study are consistent with the established epidemiology of AF. Increasing age remains the strongest risk factor for AF because of progressive atrial structural remodeling, atrial fibrosis, inflammation, oxidative stress, atrial mechanical dysfunction, and atrial cardiomyopathy, all of which promote the initiation and maintenance of the arrhythmia. The predominance of male patients in our cohort is also consistent with previous reports of hospitalized AF populations [17]. Hypertension was present in 70.2% of patients, while diabetes mellitus and ischemic heart disease were observed in 44.7% and 40.4% of patients, respectively. These findings support previous evidence demonstrating that cardiovascular and metabolic diseases frequently coexist with AF and contribute to disease progression and adverse clinical outcomes [18]. In addition to traditional cardiovascular risk factors, increasing evidence indicates that social determinants of health-including socioeconomic status, healthcare access, educational attainment, and neighborhood environment-also influence AF incidence, treatment access, and cardiovascular outcomes, highlighting the importance of comprehensive risk assessment beyond conventional clinical variables [19].

Heart failure was present in approximately one-third of patients, emphasizing the well-recognized bidirectional relationship between AF and ventricular dysfunction. AF may contribute to heart failure through loss of atrial contraction, rapid ventricular rates, and reduced cardiac output, whereas heart failure promotes atrial remodeling, left atrial enlargement, atrial mechanical dysfunction, and atrial cardiomyopathy, thereby facilitating the development and persistence of AF [20]. Persistent AF was the most common subtype, consistent with previous studies of hospitalized patients. However, because the present study did not evaluate the timing of AF diagnosis or prior rhythm-control strategies, no conclusions can be drawn regarding delayed diagnosis or access to rhythm-control interventions [21]. The mean hospital stay of 6.7 ± 3.5 days was comparable to previously published reports and likely reflects the advanced age, multiple comorbidities, and complexity of inpatient management in this population [22].

In exploratory multivariable analysis, advanced age, heart failure, chronic kidney disease, and LVEF <40% were associated with higher in-hospital mortality. These findings are biologically plausible and consistent with previous studies demonstrating that impaired cardiac function and multiple comorbidities contribute to poor short-term outcomes in hospitalized AF patients [23]. Although a previous stroke or TIA was associated with mortality in univariable analysis, it was not independently associated after multivariable adjustment. Because only 16 in-hospital deaths occurred, these regression findings should be considered exploratory and interpreted cautiously despite the model demonstrating good calibration and acceptable overall performance. Nevertheless, the identified predictors may assist clinicians in recognizing patients who require closer monitoring and more intensive management during hospitalization.

The findings have important clinical implications for tertiary-care hospitals managing patients with AF. Early recognition of high-risk individuals, optimization of anticoagulation when appropriate, effective management of concomitant heart failure and chronic kidney disease, and systematic use of validated thromboembolic and bleeding risk assessment tools may improve inpatient care. Furthermore, addressing modifiable cardiovascular risk factors together with broader social determinants of health may contribute to improved long-term outcomes in patients with AF [24].

This study has several limitations. First, its retrospective single-center design limits the generalizability of the findings and may have introduced selection bias, particularly because patients with incomplete clinical or outcome data were excluded from the analysis. Second, reliance on medical records may have resulted in incomplete or inaccurate documentation of some clinical variables. Third, because of the retrospective design, differentiation between patients admitted primarily because of AF and those who developed AF during hospitalization for another acute illness was not consistently possible. Likewise, the duration of AF before presentation, indications for electrical cardioversion, and causes of in-hospital death were not uniformly documented. Fourth, only in-hospital outcomes were evaluated; therefore, postdischarge outcomes, including recurrent hospitalization, thromboembolic events, bleeding complications, and long-term mortality, could not be assessed. Finally, although the multivariable model demonstrated good calibration and overall performance, the relatively small number of in-hospital deaths (n = 16) limited the statistical power of the model and may have increased the risk of overfitting. Therefore, the identified associations should be considered exploratory and interpreted with caution until validated in larger prospective multicenter studies.

## Conclusions

This study provides contemporary real-world data on the clinical characteristics, treatment patterns, and in-hospital outcomes of patients hospitalized with AF at a tertiary care center in Pakistan, where such evidence remains limited. The findings demonstrate that hospitalized patients with AF are predominantly older adults with multiple cardiovascular comorbidities and continue to experience substantial in-hospital morbidity despite widespread use of rate-control therapy and anticoagulation. Exploratory multivariable analysis suggested that advanced age, heart failure, chronic kidney disease, and reduced LVEF were associated with increased in-hospital mortality, although these findings should be interpreted cautiously because of the retrospective single-center design and the limited number of mortality events. These results may assist clinicians in identifying high-risk patients who could benefit from closer monitoring and optimized evidence-based management during hospitalization. Larger prospective multicenter studies are needed to validate these findings and improve risk stratification in hospitalized patients with AF.
